# Training and Support for Hybrid Closed-Loop Therapy

**DOI:** 10.1177/1932296820955168

**Published:** 2020-09-11

**Authors:** Charlotte K. Boughton, Sara Hartnell, Janet M. Allen, Julia Fuchs, Roman Hovorka

**Affiliations:** 1 Wellcome Trust-Medical Research Council Institute of Metabolic Science, University of Cambridge, Cambridge, UK; 2 Cambridge University Hospitals NHS Foundation Trust, Wolfson Diabetes and Endocrine Clinic, Cambridge, UK

**Keywords:** artificial pancreas, hybrid closed-loop, training, type 1 diabetes

## Abstract

Hybrid closed-loop therapy is an emerging technology transforming the management of type 1 diabetes (T1D). Research studies demonstrate glycemic and quality of life benefits of hybrid closed-loop therapy for people with T1D. Translating these outcomes into standard clinical practice is critical for reimbursement and improving access to this technology.

High-quality training is essential for achieving optimal outcomes with hybrid closed-loop therapy. Basic diabetes skills and tasks are as important, or even more important, with closed-loop therapy than with standard insulin therapy and need to be reiterated. Establishing expectations of hybrid closed-loop therapy clearly at the outset promotes long-term usage and optimal outcomes.

We share key aspects of training and support for users of commercially available hybrid closed-loop systems and consider who may benefit from this technology.

There is compelling evidence that closed-loop systems can improve glycemic outcomes for people with type 1 diabetes (T1D). Translating the clinical benefits observed in research settings into routine clinical practice is critical for securing reimbursement and improving access to this technology. Current commercially available closed-loop systems are hybrid closed-loop systems, which still require user interaction for mealtime boluses. In our experience in both the research and clinical setting, high-quality training is essential for optimal outcomes with hybrid closed-loop therapy. In this report, we share key aspects of training and support for users of commercially available closed-loop systems.

## Getting the Basics Right and Managing Expectations

Hybrid closed-loop systems are not “plug-in-and-play” and still require user interaction for mealtime bolusing, insulin pump set changes, and continuous glucose monitoring (CGM) device insertion (and sometimes calibrations). Neglecting or overlooking these tasks can have more significant consequences with closed-loop therapy than with standard insulin therapy, and so must be reinforced. The term “artificial pancreas” can be misunderstood to mean that the system is fully automated with no action required by the user; therefore when users start hybrid closed-loop therapy, expectations may not be met. Understanding the closed-loop system capabilities and the workload required to operate a closed-loop system is important to maintain optimal usage and therefore realize the clinical benefits.

### Carbohydrate Counting

This remains a key diabetes self-management skill. The amount and type of carbohydrate consumed is still important. Opportunities to review and optimize carbohydrate counting accuracy should be sought as in standard clinical practice. While closed-loop systems will correct some post-prandial hyperglycemia, this will not be as effective as accurate carbohydrate counting due to delays in subcutaneous insulin absorption, and closed-loop systems are not able to mitigate excessive boluses delivered for overestimated carbohydrates.

### Insulin Bolus Timing

There are important differences between closed-loop insulin delivery and standard pump therapy with regard to prandial insulin bolus timing.^1^ Closed-loop systems detect the rise in sensor glucose levels if carbohydrate ingestion is not preceded by bolus insulin delivery and automatically deliver increased insulin infusion rates to manage the glucose excursion. If the mealtime insulin bolus is given later, this may cause over-delivery of insulin and subsequent hypoglycemia if the closed-loop directed insulin is not taken into consideration. The longer the interval between meal commencement and the prandial insulin bolus being delivered, the greater the risk of hypoglycemia. Bolus calculators used during closed-loop may account only for the insulin on board from a previous bolus, and may not include any closed-loop insulin delivery in the bolus calculation, which can contribute to the risk of hypoglycemia with delayed bolusing. It is important that closed-loop users are aware of the potential risks of post-meal bolusing when starting to use closed-loop systems, and are advised to either reduce the delayed meal bolus or miss the bolus completely and allow closed-loop to manage the post-prandial glucose excursion with the consequence of higher post-prandial glucose levels.

### Treating Hypoglycemia

Closed-loop insulin delivery systems reduce the risk of hypoglycemia, but will not completely prevent it.^2^ When glucose levels approach hypoglycemia, closed-loop systems will often not have delivered any insulin for some time prior to this; therefore, sometimes less rapid-acting carbohydrate (eg, 4 g) can be used to prevent hypoglycemia or treat mild hypoglycemia (Level 1: glucose 3.0-3.9 mmol/L). This can be important for weight management. It is useful for users to review what insulin has been delivered by closed-loop to determine appropriate hypoglycemia treatment.

### Treating Hyperglycemia

**Figure 1.**
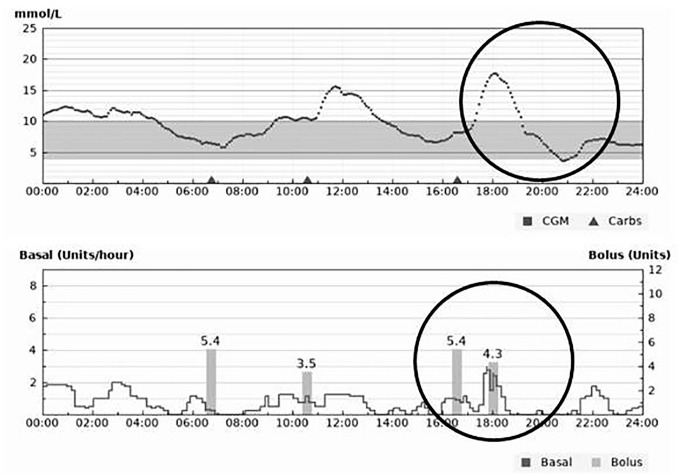
Hypoglycemia caused by manually correcting hyperglycemia during closed-loop insulin delivery. Hybrid closed-loop data over a 24-hour period with upper panel showing continuous glucose monitoring profile and lower panel showing algorithm-derived insulin delivery with manual insulin boluses.

In the event of hyperglycemia during closed-loop, the algorithm automatically increases insulin delivery to manage this; however, delays in subcutaneous insulin absorption can mean that target glucose levels are not reached immediately. If users deliver a manual correction bolus to treat hyperglycemia in addition to what the algorithm delivers, this is likely to result in hypoglycemia (Figure 1). Encouraging users to review what insulin has been delivered by closed-loop before delivering manual corrections can prevent this occurring.

If hyperglycemia is associated with ketones, users should be advised to revert to manual mode and treat with more aggressive corrections following standard sick day rules until ketones have cleared and glucose levels stabilized. If the ketonemia is thought to be caused by an infusion set issue, users should be advised to deliver corrective insulin via an insulin pen while the infusion set is changed.

### Infusion Set Changes

Closed-loop algorithms assume steady delivery and absorption of insulin from the subcutaneous tissue. Erratic insulin absorption due to lipohypertrophy or other infusion site issues can be problematic for algorithm-driven insulin delivery and can impact algorithm learning. Ensuring set changes are done every two-three days (depending on the cannula type) and infusion sites rotated will ensure optimal insulin delivery for effective closed-loop therapy.

### Suspending Insulin Delivery

It is good practice if the insulin pump is unattached from the body for more than 15 minutes to suspend insulin delivery on the pump. All insulin infused when the pump is not attached will be considered as delivered by the algorithm, which can affect algorithm estimations of active insulin and algorithm learning.

## How Does the Closed-Loop System Work?

In order for users to get the greatest benefits from closed-loop therapy, it is important that they understand how the closed-loop system works. Closed-loop systems operate in distinct ways and have individual features. Terminology also differs between closed-loop systems, which can be challenging for both healthcare professionals and users. A structured approach to closed-loop systems termed the “CARES” paradigm has been devised for use by healthcare professionals.^3^ This approach covers how each system CALCULATES insulin delivery, which parameters can be ADJUSTED, when users should REVERT to standard insulin pump settings, key EDUCATION points, and the SENSOR and SHARING capabilities of the system.

### What Are the Inputs Required by the Closed-Loop System?

Understanding what is required to initialize the closed-loop system determines how quickly someone can “go live” on closed-loop. The Medtronic 670G requires the total daily insulin dose from the past two to six days, so starting closed-loop is only possible after a “run-in” period of sensor-augmented pump therapy.^4^ The CamAPS FX system and Control-IQ require only body weight and total daily insulin dose (insulin sensitivity and active insulin time are estimated by algorithm).^5,6^

Ongoing inputs are information required to keep closed-loop in operation; the more time spent in closed-loop, the better the outcomes.^7^ All systems require real-time CGM information and input regarding carbohydrate intake and prandial insulin bolus doses based on insulin-to-carbohydrate ratios. Omission of prandial insulin boluses is possible but will significantly compromise glycemic outcomes, and in the Medtronic 670G system, can result in forced exit from the closed-loop mode if the maximum basal delivery is reached. Table 1 summarizes the ways in which closed-loop systems handle other aspects of insulin delivery.

**Table 1.** Summary of the Different Ways in Which Closed-Loop Systems Handle Insulin Delivery.

	Medtronic 670G (PID algorithm)	Medtronic 780G (PID algorithm)	CamAPS FX (MPC algorithm)	Control IQ (MPC algorithm)
What happens to basal insulin?	Overnight and between meals, the algorithm modulates the basal insulin delivery every 5-10 min based on real-time CGM data.			
What is the algorithm target glucose?	Fixed: 6.7 mmol/L	Customizable one target / 24 hours: 5.5, 6.1, or 6.7 mmol/L	Customizable at different hours of the day: 4.4-11.0 mmol/L Default 5.8 mmol/L	Fixed: 6.3-8.9 mmol/L
How is corrective insulin delivered?	Manual corrections based on HCL algorithm not programmed sensitivity factors	Automated once auto basal reaches max. Based on HCL algorithm not programmed sensitivity factors	Automated via more aggressive basal rate adjustment Option for manual corrections based on programmed sensitivity factors	Automated (60% of correction dose) if glucose predicted to exceed 10.0 mmol/L within 30 min. Option for manual corrections
How does the algorithm learn and adapt?	Based on total daily dose (TDD)	Based on total daily dose (TDD)	Adapts to prandial and diurnal patterns	Based on total daily dose (TDD)
Adjustable settings impacting on algorithm insulin delivery and features	ICR Active insulin time Exercise Mode (8.3 mmol/L)	ICR Active insulin time Exercise mode (8.3 mmol/L)	ICR Boost—algorithm more aggressive Ease Off—raises target (7.5 mmol/L) and algorithm less aggressive	ICR & ISF Basal Rates Exercise Mode (7.8-8.9 mmol/L) Sleep Mode (6.1-6.7 mmol/L)
What are the safety parameters of algorithm insulin delivery	Maximum hourly insulin delivery Maximum 4 h basal insulin delivery Minimum insulin delivery for 2.5 h Maximum basal delivery in 24 h Maximum bolus amount	Maximum hourly insulin delivery Maximum 7 h basal insulin delivery Minimum insulin delivery for 3-6 h Maximum basal delivery in 24 h Maximum bolus amount	Maximum insulin delivery in 24 h Maximum bolus amount	Maximum insulin delivery in 2 h Maximum insulin delivery in 24 h Maximum bolus amount

Note. PID, proportional–integral–derivative; MPC, model predictive control; CGM, continuous glucose monitoring; HCL, hybrid closed-loop; ICR, insulin-to-carbohydrate ratio; ISF, insulin sensitivity factor.

## Clinical Support and Optimization of Closed-Loop Therapy

### Optimization of Hybrid Closed-Loop Therapy: What Adjustments Can be Made?

- *Insulin-to-carbohydrate ratio (ICR)*. Accurate ICRs remains important for optimal glycemic benefits as this is not automated by closed-loop systems. Closed-loop systems are able to handle small inaccuracies but glycemic outcomes will likely be compromised.
- *Algorithm glucose target*. Closed-loop systems with a fixed target have limited options for temporary adjustment of the target glucose, for example, higher for exercise and lower overnight where glycemic excursions are reduced. Customizable glucose targets that can be set hour-by-hour reflect the individual user’s needs.
- *Active insulin time*. This is important if the closed-loop system requires manual insulin correction doses (eg, Medtronic 670G). The CamAPS FX algorithm estimates the individual users active insulin time behind the scenes and adapts accordingly.
- *Basal insulin infusion rates*. These are important if closed-loop operation is unavailable for example if the CGM is in warm-up. For some closed-loop systems including Control-IQ and Medtronic, basal rates strongly influence closed-loop insulin delivery.

### Managing Exercise With Closed-Loop Therapy

Individualized planning for exercise remains important with closed-loop therapy. A key difference with closed-loop is to avoid pre-exercise carbohydrate loading. The associated rise in glucose and increased closed-loop driven insulin delivery may result in hypoglycemia during exercise. It is therefore advisable to “drizzle” in carbohydrates as required before and during exercise to maintain target glucose concentration. Closed-loop systems have an option to increase the algorithm glucose target for exercise and in the CamAPS FX system, Ease Off is a functionality that not only raises the target glucose but also makes the algorithm less aggressive; it can be pre-programmed in advance. It is still best to plan the start time of exercise and consider using exercise settings for 90 minutes before, during, and after exercise. Some people may find they need to come out of closed-loop mode or even suspend insulin delivery for some forms of cardiovascular exercise.

### Alarm Burden

We proactively discuss alarm burden with people starting closed-loop therapy and advise users to minimize alarms wherever possible, especially overnight, to avoid sleep disturbances, interrupting lessons and social activities, resenting the devices, and most importantly alarm fatigue where users may become less likely to respond if experiencing persistent and frequent alarms. We suggest users set alarms for safety benefits (hypoglycemia) rather than just because they are available, and encourage people to silence alarms overnight or consider different alarm sounds for safety alarms with other alarms set to vibrate. Customization of alarms has the potential to improve usability of diabetes devices.

## Which Closed-Loop System for Which Person With Diabetes?

When considering which closed-loop system might best suit a particular person, individual choice is important. The following features vary between closed-loop systems and are therefore important to discuss with potential users to support decision-making.

- CGM features—requiring calibrations/factory cali-brated
- Device size/burden
- Remote monitoring capability
- Flexibility of the system—adjustable target glucose/algorithm which adapts to the user
- Other system features: Exercise mode/Sleep mode/Ease Off/Boost

## Who Is Suitable for Closed-Loop Therapy?

Evidence from clinical trials suggests that all populations studied have glycemic and quality of life benefits from closed-loop therapy.^2^ The strongest case can potentially be made for very young children who have the greatest day-to-day variability in insulin requirements making achieving glycemic targets very challenging,^8^ and where the quality of life benefits of closed-loop impact not just the children but also carers.^9^ There is a clear need for improved glycemic outcomes in adolescents, and more usable closed-loop systems that truly reduce the burden of diabetes management should translate the glycemic benefits observed in clinical trials into the real-world setting in this challenging population.^7^

Several large randomized controlled trials have demonstrated clinical benefits in adults and importantly for reimbursement, glycemic benefits are greatest in those with suboptimal glycemic control.^5,10,11^ The case for closed-loop therapy in pregnant women where glycemic targets are even tighter is clear, with potential benefits for both fetal and maternal outcomes.^12^ Older adults with a high burden of hypoglycemia may also benefit from this technology.^13^

Our experience in an ongoing long-term clinical study recruiting adolescents newly diagnosed with T1D has led us to feel strongly that everyone should be considered suitable for a trial of closed-loop therapy. Preconceptions about the types of people who would use technology effectively were rapidly overturned as some “technologically competent” individuals were observed interacting with the system in ways that could compromise glycemic control, and others—who healthcare professionals assumed would struggle to understand and use the technology—benefitted as they allowed the system to operate without interference.^14^ Individual, family, and psychological attributes should not be used as preselection criteria for closed-loop therapy.

## Conclusion

In order to realize the benefits of closed-loop therapy in clinical practice, high-quality training is critical. Establishing expectations of hybrid closed-loop therapy clearly at the outset promotes long-term usage and optimal outcomes. The basic diabetes skills and tasks are as important, or even more important, with hybrid closed-loop therapy than with standard insulin therapy and should be reinforced. The individual choice of closed-loop system is likely to impact usability and outcomes. Glycemic and quality of life benefits from closed-loop therapy have been demonstrated in all populations with T1D studied. Healthcare professionals must exercise caution regarding preconceptions and prejudicial assumptions to ensure fair and equitable access to closed-loop systems.
